# The Relevance of *SOCS1* Methylation and Epigenetic Therapy in Diverse Cell Populations of Hepatocellular Carcinoma

**DOI:** 10.3390/diagnostics11101825

**Published:** 2021-10-02

**Authors:** Loraine Kay D. Cabral, Peter Andrew C. Reyes, Lory S. Crocè, Claudio Tiribelli, Caecilia H. C. Sukowati

**Affiliations:** 1 Fondazione Italiana Fegato ONLUS, AREA Science Park, Campus Basovizza, 34149 Trieste, Italy; kay.cabral@fegato.it (L.K.D.C.); ctliver@fegato.it (C.T.); 2 Doctoral School in Molecular Biomedicine, University of Trieste, 34127 Trieste, Italy; 3 Hepatology Society of the Philippines, Quezon City 1105, Philippines; peterandrewr@yahoo.com; 4 Liver Unit, Clinical Department of Medical, Surgical and Health Sciences, Trieste University, 34127 Trieste, Italy; lcroce@units.it

**Keywords:** hepatocellular carcinoma, SOCS1, epigenetic therapy, DNA methylation, tumor heterogeneity

## Abstract

The suppressor of cytokine signaling 1 (*SOCS1*) is a tumor suppressor gene found to be hypermethylated in cancers. It is involved in the oncogenic transformation of cirrhotic liver tissues. Here, we investigated the clinical relevance of *SOCS1* methylation and modulation upon epigenetic therapy in diverse cellular populations of hepatocellular carcinoma (HCC). HCC clinical specimens were evaluated for *SOCS1* methylation and mRNA expression. The effect of 5-Azacytidine (5-AZA), a demethylation agent, was assessed in different subtypes of HCC cells. We demonstrated that the presence of *SOCS1* methylation was significantly higher in HCC compared to peri-HCC and non-tumoral tissues (52% vs. 13% vs. 14%, respectively, *p* < 0.001). In vitro treatment with a non-toxic concentration of 5-AZA significantly reduced DNMT1 protein expression for stromal subtype lines (83%, 73%, and 79%, for HLE, HLF, and JHH6, respectively, *p* < 0.01) compared to cancer stem cell (CSC) lines (17% and 10%, for HepG2 and Huh7, respectively), with the strongest reduction in non-tumoral IHH cells (93%, *p* < 0.001). 5-AZA modulated the *SOCS1* expression in different extents among the cells. It was restored in CSC HCC HepG2 and Huh7 more efficiently than sorafenib. This study indicated the relevance of *SOCS1* methylation in HCC and how cellular heterogeneity influences the response to epigenetic therapy.

## 1. Introduction

Hepatocellular carcinoma (HCC) is one of the most common cancers and causes of cancer-related death worldwide [1]. It has a poor prognosis, mostly caused by late diagnosis leading to limited curative treatment options. HCC is a multifactorial disease with a long-term process. It represents in its vast heterogeneity of HCC histology, molecular and cellular facets, and clinical manifestation [2,3]. HCCs molecular signatures were able to be used to classify HCCs into subclasses, and to correlate these classifications with specific biomarkers and/or prognosis [4,5,6].

Rapid advances in molecular medicine have opened new perspectives in dissecting HCC heterogeneity, including epigenetic variations. DNA methylation, one of the most studied epigenetic modifications, controls gene expression by altering the chromosomal structure, DNA conformation, DNA stability, and the function way between DNA and protein [7]. It involves the transfer of a covalent methyl group to the C5 position of the cytosine to form 5-methylcytosine by DNA methyltransferases (DNMTs) [8]. 

In HCC, DNA methylation profiling by genome-wide arrays has been explored in both clinical samples and cell lines, showing enormous variations and different clinical associations [9,10,11]. Various methylated genes have been associated with diagnosis, prognosis, and treatment options, as reviewed in [12].

Targeting DNMTs to inhibit DNA methylation has been explored as a cancer therapy. The prevention and reversal of methylation in silenced tumor suppressor genes (TSGs) in cancer can lead to restoration of its function, leading to possible suppression of malignancy. DNMT inhibitors such as 5-Azacytidine and 5-Aza-2′-deoxycytidine are being used to treat hematological malignancies [13] and other cancers [14]. However, in terms of epigenetic treatment, this inhibition of DNMTs can also lead to loss of heterozygosity and global hypomethylation leading to a general decrease in methylation activities that may affect also the normal patterns of gene regulation. Despite this concern, several studies of these DNMT inhibitors have generated outcomes that lead to the reduction of malignancy and improved survival [15]. The integration of this nucleoside must happen during the S phase of the cell cycle during the replication process. Hence, this drug can incorporate itself effectively to actively replicate tumor cells [16].

The suppressor of cytokine signaling 1 (*SOCS1*), also known as STAT-induced STAT inhibitor-1 (*SSI-1*), encodes a member of the STAT-induced STAT inhibitor. It is responsible for negative feedback regulation of the JAK–STAT pathway induced by cytokine stimulation [17]. In HCC, *SOCS1* is marked as a tumor suppressor gene. However, it is frequently silenced through epigenetic disruption. The incidence of *SOCS1* aberrant DNA methylation was around 60% in HCC tumor specimens [18,19], indicating it is a common event in HCC. The restoration of *SOCS1* upon methylation, however, suppressed HCC growth rate and anchorage-independent growth [18]. 

Even though the incidence and the clinical significance of *SOCS1* in HCC had been widely demonstrated, further knowledge is still needed to explore this biomarker in vast heterogeneous HCCs. Here, we highlight the relevance of DNA methylation of *SOCS1*, both in in vitro and clinical samples, and the effect of demethylating treatment on its regulation.

## 2. Materials and Methods

### 2.1. Samples

#### 2.1.1. Human Tissue Samples

From each patient, different portions of liver tissues composed of non-tumoral, peritumoral (peri-HCC), and neoplastic/tumoral (HCC) were collected. The diagnosis of patients was established on international criteria together with its Edmondson Steiner (ES) HCC grading, tumor parameters, laboratory results, and other clinical findings. Informed consent to participate in the study was obtained from each patient or by a legal representative and the protocol was approved by the Comitato Etico Regionale Unico of the Friuli Venezia Giulia, Prot. No. 18854. Immediately after surgery, fresh liver tissues were collected and snap-frozen in liquid nitrogen and stored at −80 °C. In parallel, liver tissues were fixed in formalin and included in the paraffin block. Fixed slices were subjected to hematoxylin and eosin (HE). The final diagnosis of patients was established in agreement based on international criteria together with its clinical findings. 

#### 2.1.2. Cell Lines

Human liver cell lines IHH, HepG2, Huh7, HLE, HLF, and JHH6 were used as in vitro models. The immortalized hepatocytes IHH were grown in DMEM-F12 medium supplemented with 10% (*v/v*) fetal bovine serum (FBS), 1% antibiotics, 1% L-glutamine, 1 µM dexamethasone, and 5 µg/mL insulin. The HepG2, Huh7, HLE, and HLF cells were grown in DMEM medium (high glucose) supplemented with 10% (*v/v*) FBS, 1% L-glutamine, and 1% antibiotics. The JHH6 cells were grown in Williams’ E medium supplemented with 10% (*v/v*) FBS, 1% L-glutamine, and 1% antibiotics. The cultures were maintained at 37 °C in a humidified 5% CO_2_ incubator and when they reached 80% confluence they were routinely expanded by 0.05% trypsin detachment.

### 2.2. Flow Cytometry

The presence of CSC surface marker antigens was detected using antibodies CD90/THY-1 (Clone 5E10, Stem Cell Technologies, VA, Canada), CD13/ANPEP (Clone WM15, Abcam, Cambridge, United Kingdom), CD133/PROM1 (clone AC133, Miltenyi Biotec GmbH, Bergisch Gladbach, Germany), CD326/EpCAM (Clone (VU-1D9, Santa Cruz, Dallas, TX, USA), CD24 (clone 32D12, Miltenyi Biotec), and CD45 (Clone 5B1, Miltenyi Biotec). After detachment, at least two million cells per mL were incubated with specific first antibodies for 60 min on ice in the dark. After two washes with PBS containing 0.5% bovine serum albumin (BSA) and 3 mM EDTA, when necessary, the cells were then incubated with fluorescence-conjugated secondary antibody for 60 min on ice in the dark. Flow cytometric analysis was performed immediately in a flow cytometer (FACSCalibur, Becton Dickinson, Franklin Lakes, NJ, USA). Ten thousand events were analyzed per sample. 

### 2.3. Isolation of Genomic DNA and Bisulfite Conversion

Genomic DNA (gDNA) extraction was performed using the EZ DNA Methylation-Direct^TM^ Kit (Zymo Research, Irvine, CA, USA), according to the manufacturer’s instructions. Briefly, tissues were lysed in digestion buffer and proteinase K for 20 min at 50 °C. Approximately 500 ng of DNA from the lysed supernatant was used for bisulfite conversion. Briefly, 200–500 ng gDNA was incubated in the conversion reagent and then treated with binding buffer in a spin column. Bisulfite-converted DNA (bcDNA) was then subjected to desulphonation and cleaned up using wash buffer. Bisulfite-converted DNA (~10 µL) was eluted and collected for methylation-specific PCR (MS-PCR).

### 2.4. Methylation-Specific PCR (MS-PCR)

**Figure 1:**
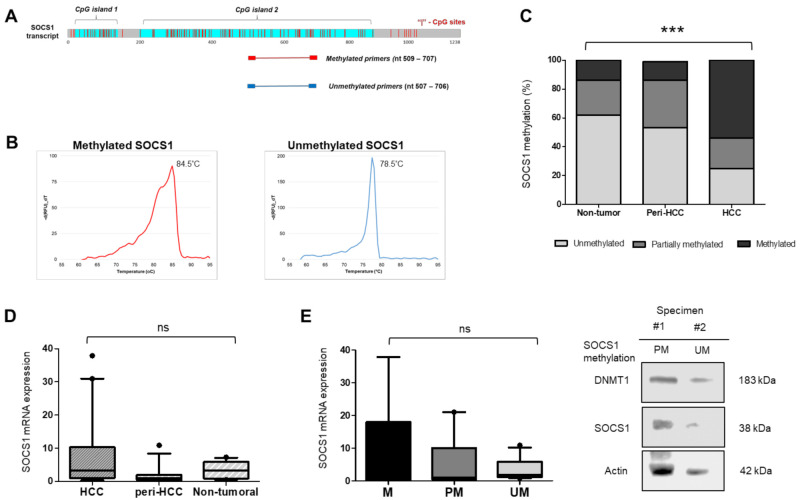
The identification of *SOCS1* methylation status in HCC clinical specimens. (**A**) The target region of *SOCS1* methylation analysis is located in CpG island 2 of *SOCS1* transcript (NM_003745.2). MS-PCR primers pairs were designed by MethPrimer 2.0 Primer Design© web tool. **(B)** The melting curve graph of MS-PCR detects specific melting peaks of methylated-specific and unmethylated-specific SOCS1 primers (M = 84.5 °C; UM = 78.5 °C). (**C**) Distribution of *SOCS1* methylation status showing a high incidence of methylated *SOCS1* among HCC tissues and unmethylated *SOCS1* in peritumoral and non-tumoral tissues. Data was presented in % value. Statistical analysis: *** *p* < 0.001 Chi-square test for all the variables in the graph. (**D**) Distribution of *SOCS1* mRNA expression among different tissue samples analyzed by quantitative RT-PCR. (**E**) No correlation between *SOCS1* methylation status and mRNA expression (PCR-based analysis, left). Representative blots of clinical specimens of DNMT1 (183 kDa) and SOCS1 (38 kDa) proteins in relation to *SOCS1* methylation status (protein blot, right). MS-PCR: methylation-specific polymerase chain reaction; M: methylated *SOCS1*; PM: partially methylated *SOCS1*; UM: unmethylated *SOCS1*; ns: not significant.

MethPrimer 2.0 Primer Design© web tool [20] was used to determine MS-PCR primers for this study, covering the region around nt 500–700 of CpG island 2 of the *SOCS1* transcript (NM_003745.2) (Figure 1a). This CpG island included exon 2 of the *SOCS1* gene. The primers covered at least 23 CpG sites and was about 200 bp in size. Primer sequences are listed in Table 1. 

Methylation-specific PCR (MS-PCR) was carried out in a 15 µL reaction volume containing 100 ng bcDNA, 1X Power-Up SYBR Master Mix (Thermo Scientific, Waltham, MA, USA), and 250 nM of methylation-specific forward and reverse primers. The presence of a methylated-SOCS1 sample was indicated by the detection of methylated-SOCS1 primer with a melting peak temperature of 84.5 °C. Accordingly, an unmethylated sample was shown by the detection of an unmethylated-SOCS1 primer with a melting peak temperature of 78.5 °C. Partially methylated samples were defined by positive detection of both primers. Purified gDNA from a human methylated and non-methylated control set (Zymo Research) was used as controls for the methylation analysis.

### 2.5. Treatment In Vitro

5-Azacytidine (5-AZA, Sigma Aldrich, St Louis, MO, USA) was used to assess the effect of demethylation in cell lines. Cells were plated with a concentration of 25,000 cells/cm^2^ (12,500 cells/mL for JHH6) for 24 h then exposed to 5-AZA with concentrations ranging from 2 uM to 5 mM for another 24 h. DMSO concentration was calculated to be 0.3% in each treatment. Cell viability was determined by 3(4,5-dimethyl thiazolyl-2)-2,5 diphenyltetrazolium assay (MTT, Sigma Aldrich). Total protein and RNA extracts were collected for proteomic and gene analysis, respectively. 

### 2.6. Western Blot Analysis

After treatment, total proteins from cells were collected using cell lysis buffer (Cell Signaling) and homogenized by scrapping and vortexing. The extract was centrifuged for 10 min at 14,000× *g* in a refrigerated microfuge. Supernatants were recovered and protein concentration was determined by the bicinchoninic acid protein assay (BCA). DNMT1 and SOCS1 protein analysis was performed by using Western blot (WB) on total protein extract obtained from treated cell lines. Proteins (10–30 µg) were size-separated by SDS–PAGE on 10% polyacrylamide gel. Electro-transferred gel onto PVDF membrane was immunoblotted with the DNMT1 antibody (Abcam, ab19905) and SOCS1 (Santa Cruz, E-9, sc-518028). Actin was used as a housekeeping protein. The peroxidase reaction was obtained by exposure of membrane in the ECL Plus WB detection system solutions (ECL Plus Western Blotting Detection Reagents, GE-Healthcare Bio-Sciences, Italy). Protein quantification was performed after the densitometric analysis of target bands vs. actin in each sample for three independent experiments. 

### 2.7. Reverse Transcription-Quantitative Real-Time PCR (RT-qPCR)

Reverse Transcription (RT) was performed to obtain cDNA from 1 µg of purified RNA with the High Capacity cDNA Reverse Transcription Kits (Applied Biosystems, Waltham, MA, USA) according to the manufacturer’s protocol. Real-time PCR was performed according to the SYBR Green Supermix protocol (Bio-Rad Laboratories, Hercules, CA, USA). PCR amplification was carried out in 15 µL reaction volume containing 25 ng cDNA, 1 × iQ5 SYBR Green Supermix, and 100–250 nM of gene-specific forward and reverse primers. The reaction was run in CFX 9600 real-time PCR system (Bio-Rad). The primer sequences are designed using Beacon Designer 7.9 Software (PREMIER Biosoft International, Palo Alto, CA, USA) for the detection of the desired gene and are listed in Table 1.

### 2.8. Statistical Analysis

Statistical significance was calculated using software GraphPad Prism version 8.0 (GraphPad Software, Inc., La Jolla, CA, USA). For human tissue samples, continuous variables of *SOCS1* methylation and *SOCS1* mRNA expression were calculated using proper tests (ANOVA, contingency chi-square or Fisher’s exact test, and t-test) following normality test. mRNA data were presented as median (95% CI). Data in vitro were obtained from at least three independent experiments and are expressed as mean ± SD. Statistical significance was set to *p*-value < 0.05 and reported as indicated here: * *p* < 0.05, ** *p* < 0.01, and *** *p* < 0.001.

## 3. Results

### 3.1. SOCS1 Methylation Status in HCC Specimens

For *SOCS1* methylation status, we developed a simple method to define the methylation status of the coding region of *SOCS1* in CpG island 2. The region flanked by the primers included at least 23 CpG sites and was about 200 bp in size (Figure 1a). Methylation analysis was based on a melting temperature of an intercalating agent SYBR Green in an MS-PCR system, thus, a reduced PCR post-analysis step of gel electrophoresis. The presence of a methylated-SOCS1 specimen was indicated by the detection of a methylated-SOCS1 primer with a melting peak temperature of 84.5 °C. In addition, an unmethylated sample was shown by the detection of an unmethylated-SOCS1 primer with a melting peak temperature of 78.5 °C. Partially methylated samples were defined by the positive detection of both primers (Figure 1b).

Total gDNA was extracted from different portions of the liver tissues composed of non-tumoral, peritumoral, and neoplastic/tumoral tissues. The *SOCS1* methylation was progressively increased along the hepatocarcinogenesis stages of the tissues (*p* < 0.001, chi-square test). Non-tumoral and peri-HCC livers were dominated by unmethylated *SOCS1* (62% and 53%, respectively), while partially methylated and methylated *SOCS1* were noticed in a small number of samples (Non-tumor: 24% and 14%; peri-HCC: 33% and 13%, respectively). In contrast with HCC, 54% of HCC samples were *SOCS1* methylated, while partially methylated and unmethylated *SOCS1* were noticed only in 21% and 25% of samples, respectively (Figure 1c). 

In relation to clinical parameters, *SOCS1* methylation status was positively associated with aging (*p* < 0.05, *t*-test). It was present more in moderate differentiation, Edmonson-Steiner histological grade 2 and grade 3 (*p* < 0.05, Chi-square), but it was not related with etiology, tumor size, Child–Turcotte–Pugh (CTP) and BCLC class, AFP level, and tumor recurrence (Table 2).

### 3.2. SOCS1 mRNA Expression Is Not Correlated with DNA Methylation

In parallel, *SOCS1* gene expression of similar sets of HCC specimens was performed by RT-qPCR. Primers for RT-qPCR were designed to cover the coding region in the CpG island 2 in exon 2, as for DNA methylation. mRNA analysis showed no significant difference in *SOCS1* mRNA expression for non-tumoral, peri-HCC, and HCC tissues (Figure 1d). *SOCS1* mRNA expression was also not associated with age, tumor size, CTP class, and tumor recurrence under 12 months post-surgery.

To check whether *SOCS1* mRNA expression was correlated with *SOCS1* methylation, we performed a comparative analysis between two parameters in the data set. Our data showed that there was no association between *SOCS1* methylation and *SOCS1* expression (Figure 1e).

### 3.3. Demethylation by 5-AZA in Different Cell Types

Based on data *in vivo* showing frequent *SOCS1* methylation in HCC clinical specimens, we assumed that demethylation of HCC cells could be a potential epigenetic strategy. However, since HCC is a very heterogeneous tumor, it is unclear whether its effect would be similar in different tumoral cells.

Here, we used six different cell lines representing cellular heterogeneity of HCC, as shown by their different phenotypes using flow cytometry. As in the literature [4,5], the cancer stem cell (CSC) subtypes HepG2 and Huh7 were determined with the presence of EpCAM+ cells where CD133+ cells were also noticed in Huh7. CSC CD24+ cells were present in HLE, HLF, and Huh7, CD13+ cells in IHH, JHH6, Huh7, and HepG2, while CD90+ cells in IHH (Table 3).

**Figure 2:**
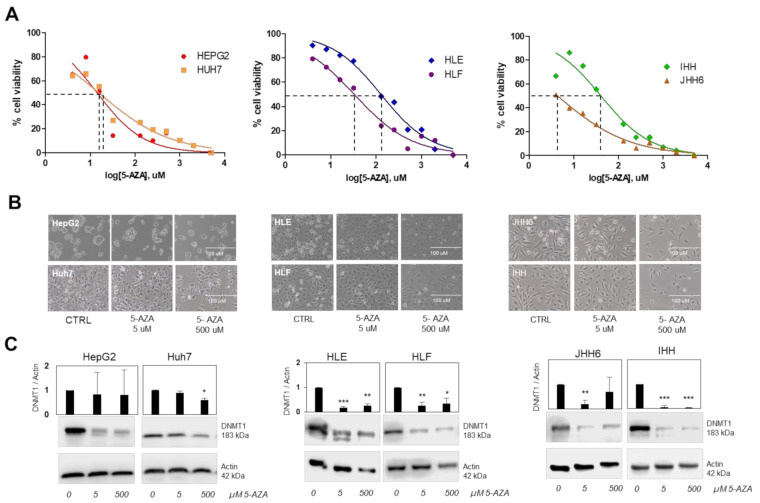
The effect of 5-Azacytidine (5-AZA) in in vitro models. (**A**) Dose response of hepatic cell lines: CSC HCC cells: HepG2 and Huh7; stromal HCC cells: HLE, HLF, and JHH6, and immortalized hepatocytes IHH. All cells were treated with 2 µM to 5 mM of 5-AZA for 24 h and cytotoxicity assay was performed by MTT test. Dashed lines show LC_50_ value for each cell line. (**B**) Cells morphology after 24 h treatment of 5 µM and 500 µM of 5-AZA. (**C**) Quantitative graphs and representative blots of DNMT1 protein expression (183 kDa) after 24 h treatment of 5 µM and 500 µM of 5-AZA. Actin (42 kDa) was used as housekeeping protein. Graphs presented as mean ± SD calculated from at least three independent experiments. Statistical analysis: * *p* < 0.05, ** *p* < 0.01, *** *p* < 0.001 using Student’s t-test relative to CTRL (0 uM) in each cell line. MTT: 3(4,5-dimethyl thiazolyl-2)-2,5 diphenyltetrazolium.

5-AZA was chosen as a demethylating drug. First, we evaluated 5-AZA cytotoxicity by MTT test to determine the non-lethal concentration 50 (LC_50_), ranging from 2 μM to 5 mM. Upon 5-AZA treatment for 24 h, the calculated LC_50_ was 128 μM for HLE, 33 μM for HLF, 41 μM for IHH, 16 μM for Huh7, 14 μM for HepG2, and 5 μM for JHH6 (Figure 2a).

Morphological analysis showed that 5-AZA of 5 μM did not alter the morphology of the cells, in contrast to 500 μM treatment (Figure 2b). Both concentrations reduced the expression of DNMT1 protein in different extents, which seemed to be correlated with cellular subtypes. The lowest DNMT1 reduction after 5 μM treatment was noticed in non-tumoral cells IHH reaching up to a 93% decrease (*p* < 0.001). The stromal HCC subtypes HLE, HLF, and JHH6 showed reductions of around 80% (83%, 73%, and 79%, respectively, *p* < 0.01). On the other hand, the CSC subtypes HepG2 and Huh7 showed rather slight decreases upon 5-AZA treatments (17% and 10%, respectively). A significant reduction was noticed only following 500 μM treatment in Huh7 of around 40% (Figure 2c).

### 3.4. SOCS1 Modulation Following Demethylation by 5-AZA

**Figure 3:**
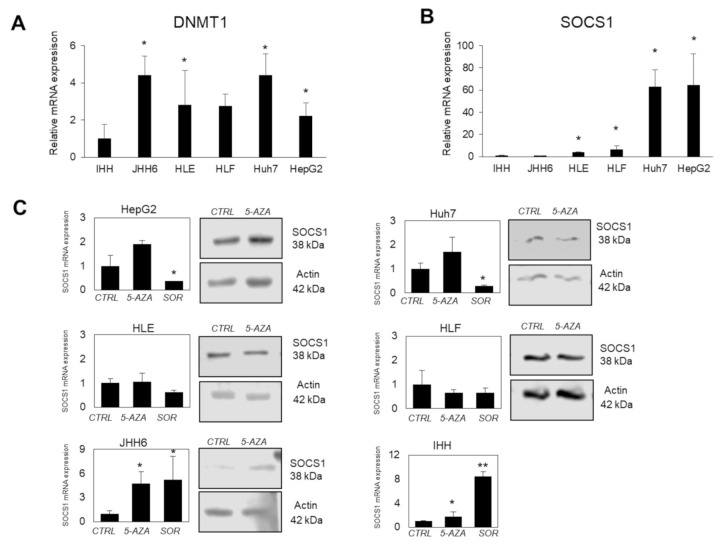
The effect of 5-Azacytidine (5-AZA) in SOCS1 expression in in vitro models. (**A**) Relative mRNA expression of *DNMT1* in HCC cell lines JHH6, HLE, HLF, Huh7, and HepG2 compared to non-tumoral IHH cells (=1.0). Statistical analysis: * *p* < 0.05 using Student’s t-test relative to IHH. (**B**) Relative mRNA expression of *SOCS1* in HCC cell lines compared to non-tumoral IHH cells (=1.0). Statistical analysis: * *p* < 0.05 using Student’s t-test relative to IHH. (**C**) mRNA expression and representative protein blot of *SOCS1* (38 kDa) in hepatic cell lines after 24 h treatment of 5 uM of 5-AZA and 50 uM of sorafenib. Actin (42 kDa) was used as housekeeping in protein blot. Statistical analysis: * *p* < 0.05, ** *p* < 0.01 Student’s t-test relative to CTRL for each cell line.

First, we checked the *DNMT1* expression among cell lines. We noticed that, compared to non-tumoral IHH cells, the *DNMT1* expressions (Figure 2c and Figure 3a) in HCC cells were significantly higher for more than 2-fold (*p* < 0.05). For *SOCS1*, *SOCS1* expression in JHH6 was comparable to that of IHH, while it was significantly higher in HLE and HLF for around 4-fold, and 6-fold, respectively (*p* < 0.05). *SOCS1* expression was noticeably much higher in CSC subtypes Huh7 and HepG2, accounting for around 60-fold for both cells (*p* < 0.05) (Figure 3b).

The 5-AZA non-toxic concentration of 5 µM was used to investigate *SOCS1* modulation in these cells. As shown in Figure 3b, following the treatment of 5 µM of 5-AZA for 24 h, the *SOCS1* mRNA expression was restored to different extents. HCC cell lines belonging to the CSC subtypes (HepG2 and Huh7) gained an approximate 2-fold increase in SOCS1 mRNA expression after 5-AZA treatment, whereas the *SOCS1* expression in stromal cells HLE and HLF was unchanged. However, a significant increase of around 4-fold was noticed in JHH6 cells (*p* < 0.05). The non-tumoral cells IHH showed a 2-fold increase in *SOCS1* expression (*p* < 0.05).

To compare, 50 µM sorafenib, an approved molecular targeted therapy against VEGFR and Raf-kinases for HCC, was also checked. Sorafenib treatment did not change the protein expression of DNMT1 (data not shown). Upon 24 h of treatment with sorafenib, in contrast to 5-AZA, the *SOCS1* expression was decreased for HepG2 and Huh7 (*p* < 0.05). Sorafenib treatment was able to restore the expression of *SOCS1* only in JHH6 and IHH cells (*p* < 0.05 and *p* < 0.01, respectively).

## 4. Discussion

Demethylation using DNA methylation inhibitors has been recognized as a potent epigenetic therapy. Indeed, 5-AZA was the first epigenetic drug to be approved by the FDA in the early 2000s for the treatment of myelodysplastic syndrome [22]. It has been used as a potential strategy to restore hypermethylation of tumor suppressor genes (TSGs) in different cancers, including in HCC cell line HepG2 [23]. Gailhouste et al. previously demonstrated the so-called epigenetic reconditioning using a non-cytotoxic dose of 5-AZA to induce HCC cells differentiation by increasing the expression of mature hepatocyte markers from the liver progenitor cancer cells. It reduced tumorigenicity and improved the cytotoxic effect of sorafenib [24]. It was in line with data in leukemia and other solid tumors where a low dose of 5-AZA has successfully reduced stem cell and CSC characteristics [25].

Here, we used a non-cytotoxic dose of 5-AZA to investigate the effect of demethylation in different HCC cellular subtypes and whether it would have a correlation with the expression of SOCS1. SOCS1 is a suppressor of cytokine signaling annotated as a TSG. During advanced carcinogenesis, downregulated *SOCS1* leads to loss of its function as a TSG and consequently activates STAT3 phosphorylation leading to malignant transformation [26]. In cancers, aberrant *SOCS1* methylation was observed in cancerous specimens, such as in multiple myeloma, pancreatic ductal adenocarcinoma, and in young patients with colorectal cancer. On the other hand, it was not noticed in healthy or non-cancerous specimens [27,28,29].

In HCC, *SOCS1* expression had an independent prognostic value where higher *SOCS1* expression in HCC predicted favorable prognosis [30]; however, abnormal *SOCS1* methylation may contribute to the pathogenesis of HCC [31]. In HCC cells LCL-PI 11 and HLE, the treatment of 5-aza-2′-deoxycytidine (5-AZA-CdR) decreased gene expressions of *DNMT1*, *DNMT3a*, and *DNMT3b* and increased *GSTP1* and *SOCS1* [32,33].

In this study, we optimized a rather simple method to determine the methylation status of the *SOCS1* gene by using a real-time MS-PCR with melting peak analysis. More importantly, we dissected the significance of *SOCS1* methylation in the European HCC cohort, where this information is still very limited. From our data, we demonstrated that the frequency of *SOCS1* methylation in HCC tissues was significantly higher than in adjacent peri-HCC and its non-tumoral tissues, in line with previously reported studies [31,34].

This study also confirmed that *SOCS1* methylation in CpG islands was not related to its mRNA expression, as had been demonstrated previously [18]. A global integrative array study of gene expression and methylation profiling in 59 HCC patients had identified 4416 CpG sites that were differentially methylated between the tumors and their adjacent non-tumorous tissues. However, only 536 of these CpG sites were associated with differences in the expression of their associated genes [10]. Studies showed that hypermethylation of CpG sites at the *SOCS1* promoter led to transcriptional silencing [31]. However, *SOCS1* methylation, either in CpG islands or promoter regions, was reported to be correlated with tumor growth and tumor size in HCC [18,35].

Since HCC is a very heterogeneous disease where DNA methylation, including for *SOCS1*, is a common event, we then evaluated whether the effect of demethylation as an epigenetic reconditioning would be effective in different cellular subtypes of HCC [4,5,6]. In this study, we observed that a non-toxic concentration of 5 µM of 5-AZA reduced significantly the expression of DNMT1 protein in non-tumoral cells IHH and stromal subtypes HLE, HLF, and JHH6, but not that of CSC subtypes HepG2 and Huh7, even though *DNMT1* basal expression in HCC lines was comparable.

Further, we showed that a non-toxic concentration of 5-AZA could restore the expression of *SOCS1*. However, its effect depended on the type of the cells. The *SOCS1* restoration upon 5-AZA was slightly effective only in CSC subtypes compared to stromal subtypes HCC, except the JHH6 cells. This data indicated that epigenetic reprogramming can be effective in CSC HCCs that are drug-resistant with high-relapse capacity after conventional treatment.

To compare, we also treated the cells with sorafenib. Sorafenib is well-known as a dual-target inhibitor targeting the serine/threonine kinase Raf and the tyrosine kinases VEGFR/PDGFR [36]. Recently, sorafenib actions have also been associated with STAT3 regulation, where IL-6/STAT3 is involved in sorafenib-resistant hepatic CSC [37]. *SOCS1* is a negative regulator of the JAK/STAT pathway, where *SOCS1* epigenetic downregulation is associated with the STAT3 activation [18,26]. A previous study showed that treatment with anti-let-7 inhibitor increased *SOCS1* mRNA expression and increased chemosensitivity to sorafenib [38].

In this study, treatment with 50 µM of sorafenib was able to increase *SOCS1* expression only in non-tumoral cells IHH and HCC cells JHH6. On the contrary, the *SOCS1* expression was significantly decreased in HepG2 and Huh7 cells, while, again, its expression was unchanged in HLE and HLF cells. It is important to notice that even though JHH6 is classified as an HCC cell line, it is not a tumorigenic cell line [39,40]. Furthermore, both IHH and JHH6 have low basal levels of *SOCS1* compared to the other cells in this study. We hypothesize that the modulation of *SOCS1* could be influenced by its non-tumorigenic characteristics.

In this study, however, we did not see any significant effects of *SOCS1* expression for HLE and HLF either upon 5-AZA or sorafenib treatments. Based on the available extensive studies on the development of targeted therapy against HCC, we predict that other agents (e.g., MET, NQO1) can be explored to reinforce the success the epigenetic therapy [41].

## 5. Conclusions

To summarize, we demonstrated that DNA methylation in TSG *SOCS1* played a significant role in hepatocarcinogenesis. Epigenetic therapy using DNA methylation inhibitor 5-AZA against HCC could efficiently reduce DNMT1 protein and might restore the *SOCS1*, but the effects might be dependent on cellular type. The prevention and reversal of *SOCS1* methylation can be a potential therapeutic target but the innate heterogeneity of HCC must still be considered.

## Figures and Tables

**Table 1:** List of primers.

Target	Sequence F (5′ → 3′)	Sequence R (5′ → 3′)	Ref.
MS-PCR			
SOCS1-methylated	ATGGTTTCGGGATTTACGAGT	TAACCACGATACGCTAACGAC	ts
SOCS1-unmethylated	AGATGGTTTTGGGATTTATGAGT	AACCACAATACACTAACAACA	ts
Gene Expression			
*ACTB*	CGCCGCCAGCTCACCATG	CACGATGGAGGGGAAGACGG	ts
*SOCS1*	CCCTTCCAGATTTGACCG	ATGGTTCCAGGCAAGTAA	ts
*DNMT1*	CCATCAGGCATTCTACCA	CGTTCTCCTTGTCTTCTCT	[21]

ts: this study; MS-PCR: methylation-specific PCR.

**Table 2:** The association between SOCS1 methylation with clinical parameters.

		*SOCS1* Methylation in HCC Tissues			
		M (%)	PM/UM (%)	All (%)	*p*
Sex [F:M]		2:10 (17:83)	2:9 (18:82)	4:19 (17:83)	0.9999
Age [year, mean ± std]		70.9 ± 7.6	64 ± 6.9	67.6 ± 7.9	0.0177 *
Tumor size [cm, mean ± std]		4.9 ± 3.7	3.9 ± 2.1	4.4 ± 3.0	0.2399
AFP [median ng/mL, min–max]		7.4 (2–5094)	7.4 (3–139)	7.4 (2–5094)	0.1129
Etiology	HCV	3 (25)	6 (55)	9 (39)	0.2310
	HBV	2 (17)	0 (0)	2 (9)	
	Metabolic	6 (50)	3 (27)	9 (39)	
	no	1 (8)	2 (18)	3 (13)	
Histological grading	ES1	1 (9)	6 (60)	7 (33)	0.0465 *
	ES2	7 (64)	3 (30)	10 (48)	
	ES3-4	3 (27)	1 (10)	4 (19)	
CTP	A	10 (83)	8 (73)	18 (78)	0.5379
	B-C	2 (17)	3 (27)	5 (22)	
BCLC	0	8 (73)	10 (91)	18 (82)	0.5865
	1–2	3 (27)	1 (9)	4 (18)	
Recurrence (m)	<12 m	3 (75)	6 (43)	9 (50)	0.5765
	>12 m	1 (25)	8 (67)	9 (50)	

M: methylated; PM: partially methylated; UM: unmethylated; HCV: hepatitis C virus; HBV: hepatitis B virus; ES: Edmonson-Steiner; CPT: Child–Turcotte–Pugh; BCLC: Barcelona Clinic Liver Cancer. Statistical analysis: * *p* < 0.05 comparing M and PM/UM group; Student’s t-test for age, tumor size, and AFP level; Chi-square test for etiology and histological grading; Fisher’s exact test for sex, CTP, BCLC stage, and recurrence.

**Table 3:** The presence of CSC marker phenotypes in hepatic cell lines by flow cytometer.

Subtypes	Cell Line	EpCAM	CD133	CD90	CD24	CD13	CD45
Hepatocytes	IHH	-	-	+/−	-	+	-
Stromal HCC	HLE	-	-	+/−	+	-	-
	HLF	-	-	+/−	+	-	-
	JHH6	-	-	-	-	+/−	-
CSC HCC	HepG2	+	-	-	-	+/−	-
	Huh7	+	+	-	+	+/−	-

## Data Availability

Data are available upon reasonable request.
